# A Review of Visual Estimation Research on Live Pig Weight

**DOI:** 10.3390/s24217093

**Published:** 2024-11-04

**Authors:** Zhaoyang Wang, Qifeng Li, Qinyang Yu, Wentai Qian, Ronghua Gao, Rong Wang, Tonghui Wu, Xuwen Li

**Affiliations:** 1 College of Information and Electrical Engineering, China Agricultural University, Beijing 100083, China; cauwzy@cau.edu.cn; 2 Research Center of Information Technology, Beijing Academy of Agriculture and Forestry Sciences, Beijing 100097, China; qwt19991104@outlook.com (W.Q.); gaorh@nercita.org.cn (R.G.); wangrong@nercita.org.cn (R.W.); wuth23@163.com (T.W.); 2209028114@stu.tjau.edu.cn (X.L.); 3 College of Informatics, Huazhong Agricultural University, Wuhan 430070, China; 4 College of Agricultural Engineering, Shanxi Agricultural University, Jinzhong 030801, China; 5 College of Computer and Information Engineering, Tianjin Agricultural University, Tianjin 300384, China

**Keywords:** weight estimation, non-contact, computer vision, image analysis, point cloud, stereoscopic vision

## Abstract

The weight of live pigs is directly related to their health, nutrition management, disease prevention and control, and the overall economic benefits to livestock enterprises. Direct weighing can induce stress responses in pigs, leading to decreased productivity. Therefore, modern livestock industries are increasingly turning to non-contact techniques for estimating pig weight, such as automated monitoring systems based on computer vision. These technologies provide continuous, real-time weight-monitoring data without disrupting the pigs’ normal activities or causing stress, thereby enhancing breeding efficiency and management levels. Two methods of pig weight estimation based on image and point cloud data are comprehensively analyzed in this paper. We first analyze the advantages and disadvantages of the two methods and then discuss the main problems and challenges in the field of pig weight estimation technology. Finally, we predict the key research areas and development directions in the future.

## 1. Introduction

The informatization and precision management of animal husbandry has become a new trend in the future development of the breeding industry [1,2,3]. This trend is of great significance as it can achieve resource conservation and environmental protection and also improve breeding efficiency and product quality. Among them, the precision feeding of pigs is crucial for the development of animal husbandry and covers multiple key aspects such as the precise ratio of feed, intelligent control of the feeding environment, and real-time monitoring of animal health. Through these measures, the weight gain speed and feed utilization efficiency of live pigs are improved. At the same time, carcass quality and production efficiency are also enhanced, which strongly promotes the development of animal husbandry in the direction of intensification, scale, and modernization and conforms to the future development trend of informatization and precision [4,5].

Pig weight monitoring occupies a crucial position in animal management. By analyzing the changes in pig weights, farmers can evaluate the reproductive potential, feed consumption, social behavior, and energy balance of pigs. The abnormal fluctuations in pig weights are important indicators for judging their health status, which is conducive to the early detection of diseases and can provide an accurate basis for predicting the optimal feeding date. In addition, pig weight is also a key parameter for assessing whether it meets the slaughter standards [6]. Real-time monitoring of animal weights has many significant advantages, such as being able to reduce losses and mortality and also playing a key role in reducing the overall breeding costs. Therefore, the weight parameter has already become a key economic factor in the growth process of pigs.

Most of the existing weight estimations are usually carried out manually. It typically takes two farm workers 3–5 min to weigh each pig on a scale [7]. Alternatively, the weight can be estimated by directly measuring the body size information of pigs. This involves measuring the size of specific body parts of pigs and then calculating the weight using a pre-established mathematical model or empirical formula. Although these methods can measure the weight of pigs, there are a series of problems associated with directly contacting pigs for measurement. Firstly, this approach requires a significant amount of labor, thus increasing costs. Secondly, it causes great stress to pigs and may even lead to injuries, which results in a decline in productivity. At the same time, workers also face risks during the operation, often leading to a situation where costs outweigh benefits.

With the development of modern information technology, technologies like computer vision and artificial intelligence have made it possible to indirectly estimate the weight of pigs [8]. Computer vision can collect pig data without stress or contact, allowing researchers to observe and analyze it carefully. By using the methods of pig body size characteristics and characteristic classification, the weight of pigs can be automatically estimated, and this technology has become an important means for estimating pig weight [9]. Extracting the body characteristics of pigs is the core aspect of estimating pig weight based on computer vision. Currently, the visual estimation of live pig weight mainly includes two methods: based on images and based on three-dimensional point clouds. Most domestic and foreign research scholars construct pig weight estimation models according to the body shape and body size characteristics of pigs. In recent years, with the rapid development of deep learning methods, algorithms such as image classification, object detection, and image segmentation based on deep learning have provided reliable technical support for data processing and analysis, and the technology of estimating pig weight based on computer vision has encountered new development opportunities. Compared with other livestock species, the breeding epidemic prevention level of pigs is higher. During the 6–8 months from the birth of piglets to slaughter, it is necessary to measure their body weight multiple times. Moreover, pigs cannot obtain the weight estimation parameters through a fixed channel like cattle and sheep. Therefore, the weight estimation methods applicable to other animals are difficult to apply to pigs.

At present, the technology of estimating the weight of pigs based on computer vision has achieved good research results. However, there are still some problems. For the weight estimation of images, there may be problems such as the influence of image acquisition angles and postures on accuracy and the influence of illumination changes on image quality and, thus, on the estimation precision. Although the above problems have been alleviated by using a depth camera and more abundant body size parameters can be obtained, there are defects such as a large amount of data, high requirements for algorithm accuracy, a long response time in the calculation process, and high algorithm complexity, which are not conducive to real-time acquisition and processing.

This research will focus on the estimation of pig weight based on computer vision, summarizing its working principles and key technologies, etc. From the aspects of data acquisition, technology research and development, and experimental verification, it will deeply analyze the main technical problems and challenges. At the same time, it will explore future research focuses and development directions, aiming to provide relevant theoretical basis and technical references for further research on accurate and efficient pig weight estimation technology in the future.

## 2. Estimating Pig Weight Using Computer Vision

As smart farming technologies continue to advance, the estimation of pig weight through computer vision has become a prominent focus in livestock research [10,11]. This technology involves capturing images or video data of pigs through a computer vision system and implementing non-contact, automated weight estimation through image processing and pattern recognition algorithms. By utilizing this method, the complexity and errors associated with manual operations are significantly reduced, while also mitigating any unnecessary stress to the pigs [12,13,14].

**Figure 1:**
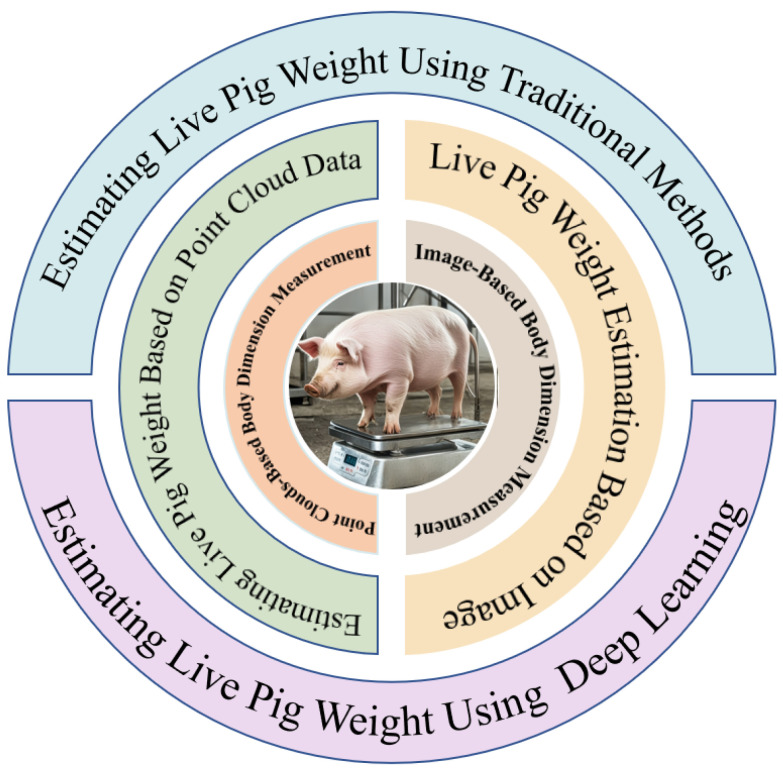
Main research elements of computer vision-based pig weight estimation.

Computer vision-based pig weight estimation primarily falls into two categories: image-based visual analysis and three-dimensional point cloud-based weight estimation [15,16,17]. Traditional image processing algorithms or deep learning algorithms are used to detect key points related to pig body dimensions from captured pig images. Subsequently, relevant body dimension information for weight estimation is extracted and categorized. Commonly used body dimension information for estimating pig weight, as shown in [18,19], includes body length, chest girth, abdominal circumference, and shoulder height, among others. Figure 1 shows the main research elements of computer vision-based pig weight estimation.

According to Panda et al. [20], body length, heart girth, waist circumference, back height, hip width, thigh circumference, neck circumference, and body height exhibit a high correlation coefficient (0.8–0.97) with weight estimation. Machebe and Ezekwe [21] conducted a study utilizing bivariate Pearson correlation analysis and other statistical methods to assess the relationship between different measurement indicators and their accuracy and reliability in predicting weight. Their findings indicated a high correlation between pig weight, body length, and heart girth. Similarly, Banik et al. [22] identified that changes in pre-weaning weight were primarily associated with front leg height (20.98%), body length (19.50%), body height (6.60%), and abdominal circumference (5.80%). Oluwole et al. [23] considered height, heart girth, body length, snout length, and hip width as influential factors in predicting pig weight. Additionally, Al Ard Khanji et al. [24] observed that body length, heart girth, and lateral abdominal distance contribute significantly to estimating pig weight.

Although the aforementioned methods have achieved good progress in weight estimation, there are also certain issues, such as measurement errors and low efficiency. Table 1 presents the body size parameters related to pig weight, weight estimation methods, and the magnitude of errors based on computer vision measurement. As can be seen from the table, although different combinations of body size parameters are used to estimate the weight of pigs, the body length of pigs, as a parameter associated with weight, is widely recognized.

In recent years, both domestic and international scholars have conducted numerous studies on pig weight estimation using computer vision, resulting in a wealth of findings. Table 2 in this paper is a review article on precision livestock farming, which compares aspects such as the research fields, research progress, and technical methods of the articles. Benjamin et al. [4] mainly introduced the application of precision livestock farming technology in monitoring and enhancing the welfare of pigs. They discussed how technological advancements, including sensors, algorithms, and machine learning, enable farmers to monitor and improve the health and behavior of pigs in real-time. Additionally, they explored the challenges and future opportunities these technologies face, as well as the crucial roles that veterinarians and practitioners play in promoting the application of technology and improving animal welfare. Rohan et al. [14] and his team mainly focused on the application of deep learning in livestock behavior recognition. Through extensive literature research and analysis, their study evaluated the effectiveness of different deep learning models and network architectures in identifying livestock behavior and also discussed the challenges encountered in practical applications. Ma [18,19] and his colleagues mainly concentrated on the techniques of three-dimensional reconstruction of livestock and the methods of measuring body size parameters. The article also pointed out the challenges faced by current technologies and provided an outlook on future development, predicting the future trends of automated livestock body size measurement technology. Based on this, this paper elaborated on the development of pig weight estimation.

Bhoj et al. [25] and Zhao et al. [26] respectively introduced the development processes of pig weight estimation and animal weight estimation. Bhoj et al. [25] placed more emphasis on introducing and comparing the methods for estimating the weight of pigs by employing different image processing strategies and machine learning models. It presented more technical details regarding the application of image processing and machine learning models in weight estimation, such as image processing algorithms, the process of model training, and the formation of databases. In contrast, this paper provided a more comprehensive overview of the main research methods, key technologies, and future development directions of the visual estimation of pig body weight. It discussed more about the overall framework and research progress of weight estimation technology, including the feasibility analysis of single-image three-dimensional reconstruction technology and the possible future trends. Zhao et al. [26] offered a comprehensive literature review, exploring the weight estimation methods for various animals and conducting an in-depth analysis of the application and challenges of deep learning in this field. However, this paper focused more specifically on the visual estimation research of live pig weight, analyzing in detail the methods based on images and three-dimensional point clouds and exploring the main challenges and future development directions in this area. In terms of technical details, Zhao et al. [26] emphasized the evolution of weight estimation methods for different animals, including the shift from traditional methods to deep learning. Meanwhile, this paper delved deeper into the specific technical implementation of live pig weight estimation, covering feature extraction methods and model construction, and provided more practical technical processes and application examples.

**Figure 2:**
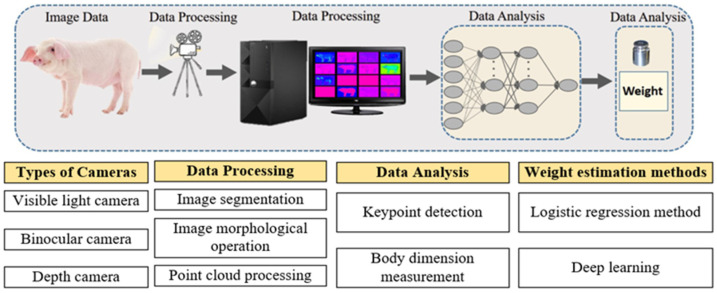
Main steps in the estimation of live pig population.

Current research in this field often focuses on stereoscopic vision, image processing, and analysis to extract pig characteristic information, and the subsequent estimation of live pig weight. The main steps involved in estimating the weight of live pigs are illustrated in Figure 2.

## 3. Live Pig Weight Estimation Based on Image

As early as 1990, Schofield et al. [27] proposed the use of digital image analysis for measuring specific areas and dimensions of live pigs in order to calculate their weight. In the early stages of research on pig weight estimation technology, the majority of studies were based on images, with researchers using visible light cameras to capture two-dimensional data of pigs for analysis. For example, relationships were established between two-dimensional parameters such as pig body area, body length, body width, and pig weight. Du et al. [28] verified the correlation between body size parameters and body weight. It can be seen from Table 3 that the parameters of body length, body width, body height, and chest depth have a relatively strong correlation, and the correlation coefficients are all greater than 0.6. The correlation between the chest circumference parameter and body weight is the largest, and the correlation coefficients are all greater than 0.9. The correlation coefficient between abdominal length and body weight is the smallest, which also confirms the rationality of using body length as a body size estimation parameter in Table 1.

A key step in estimating live pig weight using image-based techniques is the acquisition and preprocessing of images. During the estimation process, visible light cameras are typically used to collect image data of livestock in standing or walking positions. The preprocessing of the data usually involves tasks such as image segmentation, contour extraction, and image labeling, which transform the data into a suitable format for detecting and measuring body dimensions.

**Figure 3:**
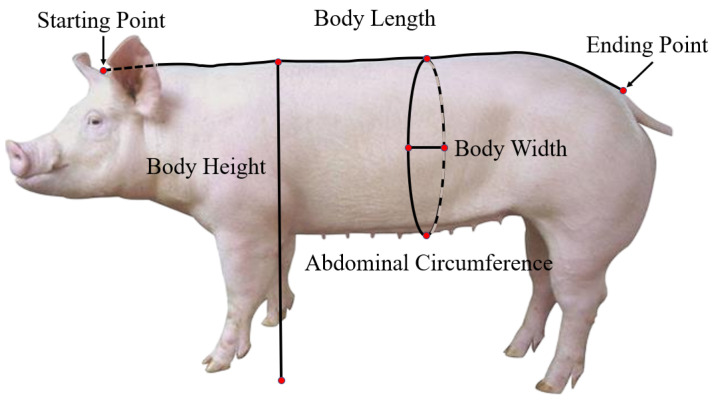
Body measurements terminologies of a pig.

The collection of livestock image data and the effectiveness of preprocessing significantly impact the accuracy of subsequent body dimension measurements. A weight prediction model is established based on the collected body dimension parameters, and the correlation between body dimensions and weight is analyzed to predict the weight of live pigs. Accurate measurement of pig body dimensions plays a crucial role in estimating live pig weight. Figure 3 illustrates the main body size parameters for pig body weight estimation.

### 3.1. Image-Based Body Dimension Measurement

Body dimension information is essential for the breeding and management of live pigs, as it not only reflects their physical size and body structure but also has significant implications for reproductive management, health monitoring, feeding optimization, and accurate weight estimation. In the early stages of machine vision-based livestock body measurement, there was a focus on exploring image information.

Whittemore et al. [29] and White et al. [30] were among the pioneering researchers who utilized machine vision technology to measure pig body dimensions. They employed visual image analysis methods to detect pigs and gather information on their back dimensions and shapes. Liu et al. [31] employed automatic threshold segmentation and morphological processing to segment the contours of live pigs, extracting external contour information from pig images. Banhazi et al. [32] developed a system using a single camera to measure pig body width, body length, and area.

Building on previous research methods, Zhang et al. [33] have developed a system that integrates livestock data collection, image data processing, and livestock body dimension measurement. This system has achieved real-time collection and processing of livestock body dimension measurements, with over 90% accuracy in detecting sheep body dimensions within a 3% error margin. Numerous studies have demonstrated the significance of precise measurement point extraction in accurately determining animal body dimensions.

Teng et al. [34] conducted research using computer vision technology to estimate live pig body dimensions and weight. They used an inflection point algorithm based on concave structures to extract key points for body length, body height, width, and hip width and calculate body dimension parameters. However, the accuracy of their method was low, and the system was not sufficiently intelligent. 

In order to measure pig body dimensions more precisely, Liu et al. [35] used envelope analysis to remove the head and tail of the pig body. They calculated key point coordinates such as body length, hip width, abdominal width, and shoulder width and used coordinate data to calculate body dimensions. The algorithm showed a smaller average relative error compared to actual measurements.

### 3.2. Weight Estimating Based on Image Body Dimension Parameters

Advancements in technology for measuring livestock body dimensions have driven the progress in estimating livestock weight. Currently, monocular cameras are predominantly utilized to capture images for detecting and analyzing relevant parameters of live pigs. Through this process, models are established to correlate the two-dimensional parameters with weight so as to estimate the weight of pigs accurately.

In the 1990s, Schofield et al. [36] carried out an analysis of pigs’ back images and found a significant correlation between weight and the projected area, as viewed from above. They developed a weight estimation model that maintained an average error controlled within 5%. In 2001, Minagawa [37] utilized stereoscopic projection technology to measure the overhead projected area and employed geometric optics principles to calculate pig body height. Subsequently, they utilized a multivariate regression equation of area and body height to estimate pig weight, achieving an average error of only 0.3 kg and a relative error of 0.8%. Doeschl et al. [38] conducted a study to investigate the changing patterns of body size and weight during the growth process of pigs. They utilized a system to continuously monitor parameters such as body length, body width, and area on the back of pigs and established a relationship model between these body size parameters, weight, and time to describe the process of pig growth transformation.

With the advancement of technology, Wang et al. [39] optimized image contour extraction methods and established a model that correlates volume parameters with weight to estimate weight in a system based on a monocular camera. This model maintains an error of 4.1%. Yang et al. [40] utilized image processing technology to calculate the actual projected area of breeding pigs and found a high correlation with weight up to 0.94. Based on this, they established a weight estimation model with a relative error of 2.8%.

Wang et al. [41] established a pig weight detection system based on a monocular camera. They selected the projected area of the pig’s back for estimating the pig’s weight. Meanwhile, to reduce the influence of light, an improved wide-range boundary detection algorithm was adopted, ensuring that the segmentation effect was not affected by light. Kaewtapee et al. [42] used a camera to obtain the back image of a pig. Through morphological operations, they calculated the proportion of live pig pixels to the total area and used the methods of regression analysis and artificial neural network (ANN) to establish a live pig weight estimation model.

Wu et al. [43] collected data using a monocular camera and employed the improved PBAS foreground detection algorithm and Canny edge detection algorithm to extract pig contour features. They also used rectangular red labels placed on the pig’s back and side to calculate body dimensions, establishing an evaluation model that predicts the weight of sows with an accuracy of over 95.5% in complex pig house environments. 

As livestock body dimension measurement technology continues to advance, the measurement accuracy of numerous studies has reached levels suitable for commercial application. Banhazi [44] developed a system based on a single camera to detect pig body width, length, and area. These parameters were combined to estimate weight with an average error of 1.18 kg. In order to further improve the portability and simplicity of live pig weight estimation equipment, Gaganpreet et al. [45] devised a novel, portable, and user-friendly method using the smartphone image measurement application “On 3D Camera Measure.” This method involves taking overhead and side views of pigs in order to measure their body dimensions. The researchers applied this technique specifically to Ghoongroo pigs (a breed from India) and successfully predicted their weight. The goal of this method is to achieve non-contact measurement of pigs.

Cunha et al. [46] introduced a method for predicting the weight of pigs by using computer vision and machine learning algorithms with the aid of two-dimensional images. The researchers developed a multiple linear regression-based mathematical model that can automatically extract morphometric data from images, such as dorsal length, thoracic width, abdominal width, dorsal area, and dorsal circumference, for predicting the weight of pigs. The experimental results show that the average error of weight estimation is 2.39 kg, and the $R^{2}$ value is 0.88. This research provides a low-cost and efficient weight-monitoring tool for pig production, especially suitable for small farms with a relatively low technical level. Wan et al. [47] used a monocular vision method based on the improved EfficientVit-C model for rapidly, accurately, and non-invasively estimating the weight of pigs. This method, through image segmentation technology combined with depth estimation and an adaptive regression network, can effectively estimate the weight of pigs using only a single camera under different lighting conditions. Table 4 shows the estimation of pig weight based on image parameters.

As can be seen from Table 4, whether it is a single-camera shot or a multi-camera shot, a top view is indispensable. The top view contains parameters that are highly relevant to weight, such as body length and body width. Although there are other features used for weight estimation in Table 4, these features also have a certain connection with parameters like body length and body width.

The previously mentioned methods for identification are based on estimating the weight of pigs from images. This means that the angle and posture of the pigs in the images significantly impact the accuracy of weight estimation. If a pig’s posture is not ideal or if the shooting angle is improper, it could lead to misinterpretation of body features, thereby affecting the results of body dimension and weight estimation [48]. According to Table 4, it is known that the change of illumination has a certain influence on the estimation of the weight of live pigs. Uneven or inappropriate illumination may lead to the appearance of shadows or overexposure in the image, causing the boundary of the pig’s body shape to be unclear and, thus, affecting the accuracy of body size and weight estimation.

### 3.3. Estimating Body Dimensions and Weight Using Binocular Stereoscopic Vision

Although using a single camera can effectively estimate the body dimensions and weight of pigs, the parameters for measured body dimensions are somewhat limited. In order to increase the range of measurement items for livestock body dimensions, some studies have captured multi-angle views of livestock and simultaneously measured multiple body dimension parameters. 

With the advent of new concepts and technologies, such as 3D modeling and 3D printing, there has been a gradual shift in focus from two-dimensional planes to three-dimensional spaces. Research has demonstrated that three-dimensional parameters, including volume, cross-sectional area, and three-dimensional measurement points, more accurately represent animal phenotypes and exhibit a high correlation with their weight. In contrast to two-dimensional images, three-dimensional measurements can directly capture live pigs’ height information, therefore overcoming the limitations of two-dimensional estimation methods and improving the precision of prediction models. 

The current method is based on binocular stereoscopic vision technology, which employs two image sensors to capture RGB images of the pig’s body. This technique utilizes vision technology to map image points onto a three-dimensional coordinate system, extracting three-dimensional features of the pig’s contour and back area. It also measures pig parameters such as body length, body width, and rump height. Furthermore, it establishes related weight prediction models in order to estimate the weight of pigs.

Binocular stereoscopic vision technology offers rapid and accurate advantages in three-dimensional measurement applications, enabling the authentic reproduction of the three-dimensional structure of objects.

Teng et al. [49] have made significant progress in the study of pig body dimension detection and weight estimation through the use of binocular vision technology. Si et al. [50] positioned cameras at a height of approximately 170 cm above the ground and 110 cm to the side of the corridor, capturing both overhead and side views of 103 pigs. They proposed an algorithm for detecting the ideal posture of pigs, which adjusts the pig’s body to a horizontal orientation, can identify the position of the head and tail, and assesses whether the head position is skewed. The results showed that the average accuracy of body width measurement was 95.5%, body height measurement was 96.3%, and body length measurement was 97.3%. Shi et al. [51] developed a mobile measurement system based on the LabVIEW platform for automatically measuring the weight composition parameters of pigs, such as body length, body width, body height, hip width, and hip height, in large-scale farms. The system uses a binocular camera to capture the back image of the pig and estimates the weight composition of the pig through image processing technology. The experimental results show that the system has high accuracy and reliability compared with the manual measurement method, providing a non-contact and efficient measurement method, which is beneficial for improving the production quality and management efficiency of the farm. Xue et al. [52] proposed employing a passive vision-based binocular stereoscopic vision method that automatically acquires data while the animal remains stationary.

During the process of camera calibration, a methodology integrating Bayesian principles, SIFT, and epipolar geometry was employed to tackle the two primary and challenging issues of camera calibration and stereo matching in binocular stereoscopic vision. This approach effectively mitigates the adverse effects of environmental factors on weight estimation. However, it is worth noting that this method imposes stringent requirements on the animal’s activity state and posture.

Shi et al. [53] employed a binocular stereoscopic vision system for capturing pig images and calculated the body dimensions of length and body height with average errors of 1.88 cm and 0.81 cm, respectively. They also utilized image processing to estimate the weight of the pigs, achieving an error rate of 1.759 kg. These findings validate the potential of binocular stereoscopic vision systems in computing three-dimensional data. The correlation between dorsal area and weight in pigs not only serves as a method for weight estimation but also allows for post-hoc evaluation of body shape.

Binocular stereoscopic vision technology has rapid and accurate advantages in three-dimensional measurement applications and can authentically reproduce the three-dimensional structure of objects. However, its configuration and calibration are relatively complex, disparity calculation is resource-intensive, and image quality is significantly affected by lighting, leading to bottlenecks in the accuracy of weight estimation.

## 4. Estimating Live Pig Weight Based on Point Cloud Data

With the widespread adoption of depth cameras and advancements in related scientific technologies, point cloud technology has been increasingly utilized. This technology relies on three-dimensional coordinate point sets captured by 3D scanning devices, which record detailed physical characteristics of an object’s surface, including, but not limited to, three-dimensional positions (X, Y, Z coordinates), color, classification, intensity values, and temporal information [54]. The popularity of consumer-grade 3D cameras such as Microsoft Kinect, Asus Xtion sensor, and Intel Real Sense has led researchers to explore the use of 3D point cloud data for measuring animal body dimensions and estimating weight [55]. As a result, point cloud technology has become a key research direction in the fields of computer graphics and computer vision. Considerable progress has been made in using 3D point clouds to estimate the weight of live pigs.

Unlike image-based pig weight estimation, point cloud-based pig weight estimation involves the collection of livestock image data using depth cameras or 3D scanners. Compared to traditional color images, depth cameras can measure the distance from the camera to the object, allowing for the creation of a three-dimensional model of the livestock based on depth information. This enhances the precision of livestock body dimension measurements. Point cloud data is voluminous and complex in data type; therefore, preprocessing of livestock point cloud data is currently a focus in research on point cloud-based livestock body dimension measurements. Through preprocessing operations such as object detection and segmentation, point cloud simplification and completion, multi-view point cloud registration, and normalization of livestock posture, a complete, edge-smoothed, uniformly oriented livestock target image can be obtained. This effectively improves the accuracy of livestock body dimension measurements.

Detection and segmentation of livestock targets are the first steps in preprocessing image data. The quality of object detection and segmentation significantly impacts subsequent data processing [56]. Once the livestock target point cloud has been detected and segmented, simplifying the livestock point cloud can effectively eliminate redundant data and retain only the key feature-containing point clouds, thus enhancing data processing efficiency. When capturing side-view image data of livestock, partial loss of data often occurs due to obstructions from pen railings, which affects the accuracy of key point localization and measurement in livestock body dimension measurements.

The general process of multi-view point cloud registration consists in using depth sensors to collect the surface depth information of livestock. After data preprocessing and livestock target extraction, methods such as point cloud registration algorithms and calibration object registration are employed to conduct stereoscopic matching of the data obtained from multiple sensors. These methods work by aligning and matching the point clouds from different sensors to ensure accurate integration of the data. Ultimately, a three-dimensional reconstruction model of the livestock’s body is obtained. After obtaining the accurate three-dimensional reconstruction model, the next crucial step is to perform posture normalization, which entails rotating and translating the segmented livestock model into a predetermined global coordinate system. This ensures a consistent orientation for the livestock model while simplifying the complexity of the algorithm for measuring the dimensions of the livestock’s body.

**Figure 4:**
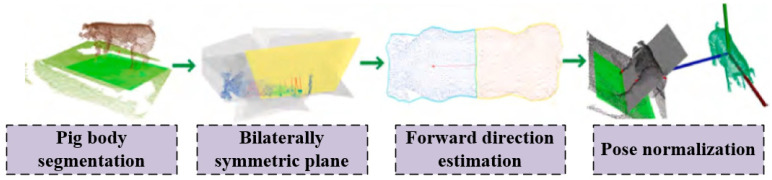
Schematic of point cloud-based normalization of livestock posture.

Figure 4 illustrates the process of normalizing livestock posture. The body dimension parameters of the collected livestock are calculated from the point cloud data, a weight prediction model is established, and the correlation between body dimensions and weight is analyzed to predict the weight of live pigs.

### 4.1. Measuring Live Pig Dimensions Using Point Clouds

Point cloud information enables the reconstruction of livestock’s actual dimensions, such as back area, body length, and body width, thereby enhancing the accuracy of weight estimation. The three-dimensional structure provides a more intuitive and clear presentation of the animal’s physical state. In recent years, 3D reconstruction has emerged as a prominent research focus in computer vision. The reconstruction of animal body type models and calculation of their body dimensions are widely applied in non-contact weight estimation methods. 

Wang et al. [57] proposed a rotation normalization technique for extracting measurement points on a pig’s body. This method effectively controls measurement errors within 16 mm and significantly simplifies the data collection process. Moreover, Wang et al. [58] developed a single-viewpoint point cloud mirroring measurement technique. By using a single depth camera, they can reconstruct a complete pig point cloud even under partially occluded conditions. This approach enables the extraction of parameters such as body length, rump width, and rump height with detection errors of 5.0%, 7.4%, and 5.7%, respectively.

In order to obtain more comprehensive point cloud information, Yin et al. [59] used a Kinect v2 camera to gather partial point cloud data of pigs from three different perspectives. Through the application of a contour continuity-based point cloud registration fusion technique, they were able to reconstruct the three-dimensional shape of the pig and estimate key body dimensions such as body height, length, width, and girth. This approach facilitated a thorough analysis of the pig’s physical characteristics.

Guo et al. [60] utilized an Xtion Pro camera to gather point cloud data and devised a sphere calibration algorithm based on Random Sample Consensus in order to automatically extract key points and body dimensions. This approach enabled the automatic and swift registration of point cloud data, with overall errors being controlled within 4%. Consequently, this validates the efficacy methods like point cloud registration algorithms and calibration object registration, which are employed to conduct stereoscopic matching of the data of the Xtion Pro camera for measuring body dimensions. The optimization of point cloud data and the improvement of computational efficiency are crucial issues in point cloud body measurement. Qin et al. [61] have developed a 3D measurement system based on dual Kinect cameras, utilizing an octree-based K-means clustering algorithm to streamline point cloud data and enhance computational efficiency. This approach is combined with pig body features for measuring body dimensions, resulting in a relative error of 3.1% for five main body dimensions.

Guo et al. [62] have developed an interactive livestock body measurement software based on the feasibility of using depth cameras to measure livestock body dimensions. This software offers a semi-automated suite of tools for loading, rendering, segmenting, normalizing posture, and measuring livestock body dimensions based on body point clouds. It provides notable data processing precision and potential for commercial application. However, this interactive body measurement software still relies on the manual selection of measurement points on the livestock’s body despite achieving some level of automated measurement of livestock body dimensions. Hu et al. [63] proposed an automated method for pig body dimension measurement using an improved PointNet++ point cloud segmentation model to rapidly locate measurement points and accurately obtain body dimensions. This method involves segmenting the overall pig point cloud into different parts, such as the head, ears, trunk, limbs, and tail, in order to locate key measurement points within the segmented parts. Wang et al. [64] developed a portable automatic measurement system based on two depth cameras for measuring the body size parameters of pigs, such as body width, hip width, and body height. The system realizes precise measurement by means of capturing point clouds, registering point clouds, removing background point clouds, extracting pig point clouds, normalizing postures, and applying morphological constraints.

The accuracy and stability of this method were validated by comparing it with manual measurement results. Menesatti et al. [65] utilized single-viewpoint depth images to capture livestock backs and extracted three-dimensional point cloud data from them. Salau et al. [66,67] employed a multi-view approach to capture depth images of different parts of livestock and obtained precise body dimension information through registration and fusion of the point cloud data. Du et al. [68] presented an automated method for measuring livestock body dimensions using multiple-depth cameras and key point detection. This approach involves merging 2D images with 3D point cloud data to identify key points on high-resolution RGB images, which are then projected onto the livestock’s 3D point cloud surface. The method provides accurate measurements of body dimensions for livestock such as cattle and pigs, while also improving measurement efficiency and reducing stress on the animals. Experimental results have confirmed its accuracy and robustness when compared to traditional manual measurements.

Luo et al. [69] proposed a method for animal body measurement based on statistical shape models. This approach involves fitting the statistical shape model of an animal to point cloud data from livestock and then extracting body measurements from the reconstructed mesh. The method is designed to tackle the challenges posed by point cloud data loss and inconsistency resulting from livestock movement in precision livestock farming. Validation of the proposed method was conducted on two common types of livestock, namely cattle and pigs, yielding an overall estimation accuracy of 91.95% for cattle and 87.63% for pigs. Lei et al. [70] developed a non-contact sensing system. This system uses a depth camera to obtain the body data of pigs and enhances the quality of 3D reconstruction by means of data preprocessing and normal estimation methods. The experimental results indicate that this technology can rapidly and accurately reconstruct the 3D model of pigs, with the measurement errors of chest circumference and hip circumference being only 3.55% and 2.83%, respectively. This research offers a new monitoring tool for the pig breeding industry and is conducive to improving the efficiency of breeding management and the health level of pigs. Table 5 presents the measurement of pig body size parameters based on three-dimensional point clouds, summarizing the number of cameras, shooting angles, the age of pigs, body size parameters, etc. Through comparison with the weight estimation parameters in Table 4, it was discovered that body height and chest circumference are accurately measured multiple times, and these two parameters are highly correlated with the estimation of weight.

### 4.2. Estimating Live Pig Weight Based on Point Clouds

Compared to traditional image-based methods, point cloud-based techniques enable the measurement of livestock features such as waist and hip circumferences. This provides richer body dimension parameters and enhances detection accuracy, ultimately improving the accuracy of livestock weight estimation results. 

Condotta et al. [71] employed depth images to predict the weight of live animals, specifically focusing on pigs at growth and fattening stages. They developed an algorithm to process these images in MATLAB 7.6 software by using depth images obtained from Kinect sensors to capture the volume of pigs. The research also investigated the impact of different genders and breeds on prediction accuracy, revealing that the overall model demonstrated commendable predictive capabilities. Cang et al. [72] proposed an intelligent method for estimating pig weight using deep learning. They designed a deep neural network that takes top-view depth images of a pig’s back as input and outputs weight estimates. This network, based on the Faster-RCNN architecture, incorporates a regression branch to integrate pig identification, localization, and weight estimation. He et al. [73] used 3D imaging and a regression network for non-contact weight measurement of pigs. The study introduced an image enhancement preprocessing algorithm and a BotNet-based regression network to predict pig weight accurately. The network, enhanced with convolution and multi-head self-attention (MHSA) branches in parallel fully connected layers, achieved a mean absolute error (*MAE*) of 6.366 kg on the test image set. Arthur et al. [74] proposed a system that uses a 3D camera to acquire pig body measurement data and predict their weight automatically. The system is capable of predicting pig weight online without the need for manual intervention, achieving this by extracting image features and measuring shape descriptors. The study conducted comparisons between datasets containing animals from different age categories versus those consisting solely of mature pigs in order to investigate the impact of dataset selection on the accuracy of weight prediction. Li et al. [75] conducted experiments on 50 pigs’ body parameters as variables for regression analysis models. Their study demonstrated the accuracy and reliability of the Kinect v2 camera in measuring body shape and estimating live weight. The final ridge regression model achieved high accuracy, with an $R^{2}$ value of 0.958 and a mean absolute error (*MAE*) of 2.961 kg, showcasing its effectiveness in this application.

Na et al. [76] developed a pig weight prediction system based on Raspberry Pi, which consists of three components: collecting pig image data, predicting pig weight, and visualizing prediction results. Initially, 3D depth camera images of pigs are captured. Then, using a Raspberry Pi module, pigs are segmented from the input images, and features are extracted from the segmented images to predict their weight. The system’s performance is trained using a 3D sensor dataset collected from a specific farm and validated using independent data, demonstrating relatively good predictive capability. Nguyen et al. [77] conducted a study in which they utilized a handheld, portable RGB-D imaging system to estimate the weight of pigs. The researchers collected RGB-D data from fattening pigs of various weights and generated a point cloud for each pig. They then employed latent features from a 3D generative model to predict the weight of the pigs. Kwon et al. [78] proposed a deep learning-based method for rapidly reconstructing mesh models from pig point cloud data and extracting various measurements to estimate pig weight in real-time. The data was collected using a point cloud acquisition system, and a deep neural network (DNN) model was developed to predict the weight. Experimental results showed high accuracy, with prediction errors on the test dataset of only 4.89 kg, accounting for 2.11% of pig weight. This research provides a new technological approach for rapidly and accurately estimating livestock weight in the livestock industry. 

Selle et al. [79] introduced a three-dimensional data modeling method for pig breeding. By constructing a statistical shape model, the size, morphology, and posture changes of pigs can be quantitatively and intuitively analyzed. Through linear regression analysis, with only body volume as a predictor variable, an accurate prediction of pig weight has been achieved. Table 6, which is about the estimation of live pig weight based on three-dimensional point clouds, summarizes the number of cameras, shooting angles, weight estimation parameters, weight estimation methods, etc. It can be seen that during data collection, the angles of the cameras are mostly top–down, or there is the participation of top–down cameras. Compared with the weight estimation parameters in Table 4, many parameters related to weight have been added, which makes up for the deficiencies of two -dimensional images.

While the use of 3D point clouds for measuring and estimating the weight of livestock has improved detection accuracy compared to traditional two-dimensional images, it is important to note that the large volume of data generated by 3D point clouds requires high algorithmic precision. As a result, the computation process leads to longer response times and higher algorithmic complexity, which is not conducive to real-time data collection and processing.

## 5. Methods of Estimating Live Pig Weight

To estimate the weight of live pigs, body feature parameters are extracted from images, and mathematical regression methods are used to predict their weight. This process includes traditional stages based on machine learning and stages based on deep learning. 

In the traditional machine learning stage, body feature parameters are either measured manually or extracted from collected animal images using algorithms and software. Parameters that show a high correlation with animal weight are selected for use in estimating the animal’s weight through mathematical regression methods such as linear regression, nonlinear regression, or machine learning. In the deep learning stage, specific animal images or video segments serve as inputs for the deep learning model. Through training computations performed by deep neural networks, the model outputs the animal’s weight or other target parameters.

Once the model for estimating live pig weight is determined, it is crucial to analyze its accuracy and relevance. The evaluation aims to compare differences between different prediction models for the same species or the applicability and performance differences of the same model on different species. Commonly used evaluation metrics in animal weight estimation tasks include mean squared error (*MSE*), mean absolute error (*MAE*), root mean squared error (*RMSE*), mean absolute percentage error (*MAPE*), and the coefficient of determination $R^{2}$. *MSE* represents the effectiveness of the model fit; the smaller the *MSE*, the better the model fit and the more accurate the model’s predictions. The formula for *MSE* is as follows:

(1) $$MSE=\frac{1}{n}\sum_{i=1}^{n}{\left(Y_{i}-{\tilde{Y}}_{i}\right)}^{2}$$

Mean absolute error (*MAE*) is the average of absolute errors used to assess the model’s fit on given data. Its magnitude also reflects the performance of the live pig weight model. The formula for *MAE* is as follows:

(2) $$MAE=\frac{1}{n}\sum_{i=1}^{n}\left|Y_{i}-{\tilde{Y}}_{i}\right|$$

Root mean squared error (*RMSE*) is a commonly used metric to measure the differences between the predicted values of a model and the actual observed values. It is used to evaluate how well a model fits the given data. The formula for *RMSE* is as follows:

(3) $$RMSE=\sqrt{\frac{1}{n}\sum_{i=1}^{n}{\left(Y_{i}-{\tilde{Y}}_{i}\right)}^{2}}$$

Mean absolute percentage error (*MAPE*) is commonly used to evaluate the discrepancy between predicted values and actual values. The formula for *MAPE* is as follows:

(4) $$MAPE=\frac{1}{n}\sum_{i=1}^{n}\left|\frac{Y_{i}-{\tilde{Y}}_{i}}{Y_{i}}\right|\times 100\%$$

$R^{2}$, also known as the coefficient of determination, is a statistical measure used to evaluate the goodness of fit of a regression model. The formula for $R^{2}$ is as follows:

(5) $$R^{2}=1-\frac{\sum_{i=1}^{n}{\left(Y_{i}-{\tilde{Y}}_{i}\right)}^{2}}{\sum_{i=1}^{n}{\left(Y_{i}-\bar{Y}\right)}^{2}}$$

In the formula above, n represents the number of samples, ${\tilde{Y}}_{i}$ is the predicted value for the *i*-th sample, $Y_{i}$ is the actual value of the *i*-th sample, and $\bar{Y}$ denotes the average of the predicted values.

### 5.1. Estimating Live Pig Weight Using Traditional Methods

**Figure 5:**
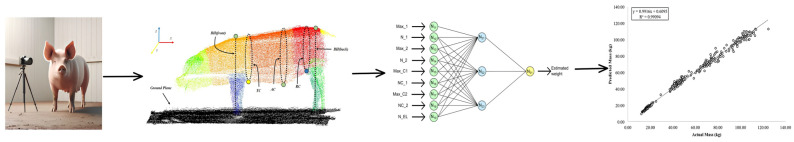
Workflow for estimating live pig weight using traditional methods.

Traditional machine learning methods for estimating pig weight are known for their simplicity, wide applicability, and ease of use. With advancements in mathematical regression techniques, feature parameters have progressed from one-dimensional to multidimensional representations. The integration of machine learning methods into the regression prediction process can significantly enhance prediction accuracy. As shown in Figure 5, the process of estimating live pig weight using traditional methods begins with image collection and preprocessing, followed by the extraction of weight-related features. Subsequently, the optimal prediction model is utilized to predict the weight of live pigs, leading to obtaining the estimated weight of the pigs.

In the early stages of weight estimation, scholars often used regression techniques to estimate the weight of live pigs. Schofield [36], Kashiha [80], and Shi et al. [81] employed linear regression to estimate pig weight. Multivariate linear regression has been utilized for animal weight estimation to improve the accuracy of weight estimation further. Sungirai et al. [82] used a stepwise multivariate linear regression method to build a weight estimation model, employing linear body measurements such as body length and chest girth to estimate live pig weight. Alenyoregue [83] analyzed the relationship between these linear body measurements and actual weight using simple linear regression and multivariate linear regression methods. To further enhance the predictive accuracy of multivariate regression models for live pig weight, Al Ard Khanji et al. [24] developed new multivariate regression models and found that the new models were more accurate than previous ones. The use of nonlinear models has further improved the precision of livestock weight estimation; Wang et al. [12] found that nonlinear models were more effective than linear models for estimating pig weight. Szyndler-Nedza et al. [84] used both linear and nonlinear models to estimate pig weight, finding that the nonlinear estimates were more accurate. Wongsriworaphon et al. [85] developed a nonlinear weight estimation method based on digital image analysis, solving the inconveniences of traditional weighing methods in farm environments and providing farms with a non-invasive, practical method for measuring weight. Jun et al. [86] fully utilized the latest advancements in machine learning technology when building their estimation models, developing new nonlinear weight estimation models. In addition to the traditional pig body area as a primary feature parameter, they introduced two new features, curvature and deviation, which are related to the pig’s posture and help more accurately adjust the weight estimation.

Pezzuolo et al. [87] utilized a depth camera for non-contact measurement of pig body sizes and developed linear and nonlinear models to estimate the weight of pigs. The research indicates that the estimation results of the nonlinear model are highly correlated with the directly measured weight, and the average absolute error is reduced by over 40% compared to manual measurement, demonstrating the potential and accuracy of this method in monitoring pig growth and health. Ruchay et al. [88] predicted the live weight of Duroc, Landrace, and Yorkshire pigs by comparing multiple machine learning methods. The research revealed that the Stacking Regressor model performed best on the test dataset, with an average absolute error (*MAE*) of 4.331 kg and an average absolute percentage error (*MAPE*) of 4.296%, suggesting that machine learning technology has high accuracy and application potential in predicting the live weight of pigs. Preethi et al. [89] employed artificial neural networks and nonlinear regression models to predict the weight of piglets at different growth stages based on 24 linear body size parameters. The research discovered that specific combinations of body size parameters, such as chest circumference, body length, and abdominal circumference, exhibited a high correlation when predicting the weight of piglets. The research findings are beneficial for estimating the weight of pigs through simple body size measurements in a farm environment lacking direct weighing conditions.

Tu et al. [90] developed a computer vision-based system, vision analysis and prediction (VAP), for estimating the weight of pigs in a slaughterhouse pen. The system developed a visual algorithm to segment the image and identify the pig area and then utilized statistical analysis and linear regression to predict the weight of the pig. Jiang et al. [91] adopted an integrated regression model method to estimate the weight of pigs. By using an Azure Kinect DK camera to collect the back images of pigs and then employing deep learning and machine learning techniques to extract and analyze image features, ultimately achieving a high-precision prediction of the weight of pigs. Lin et al. [92] utilized an improved Poisson reconstruction algorithm to estimate the weight from the three-dimensional point cloud data of pigs. The weight of the pigs was estimated by collecting point cloud data of 479 pigs from multiple angles and combining steps such as point cloud preprocessing, normal vector estimation, and mesh model reconstruction to obtain the volume of pigs, and finally, using a linear regression model. 

Table 7 presents the estimation of pig weight using traditional methods, summarizing image acquisition devices, shooting angles, weight estimation parameters, weight estimation methods, etc. It can be observed that with the passage of time, depth cameras have gradually replaced the use of visible light cameras, and the quantity of weight estimation parameters has also increased.

While machine learning methods have straightforward logical principles, facilitating easy decision-making and high efficiency, traditional machine learning algorithms are generally less accurate in comparison to deep learning models. Furthermore, deep learning methods typically demonstrate strong generalization across different species and domestic animals in various environments.

### 5.2. Deep Learning-Based Weight Estimation

**Figure 6:**
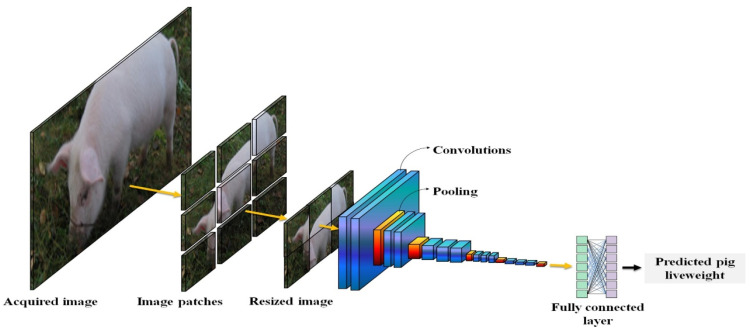
The process of weight estimation based on deep learning.

Through continuous exploration and experimentation by experts and scholars, end-to-end deep learning methods have been developed for estimating animal weight. Deep learning algorithms can accurately and efficiently extract high-value features from high-dimensional complex data, yielding results that are more accurate and reliable than those obtained with traditional machine learning methods. Figure 6 shows the process of estimating body weight based on deep learning.

Meckbach et al. [93] collected the depth image data of over 400 pigs at different growth stages and combined it with the actual weights of these pigs to train a deep learning model. This model is capable of directly learning from the images and extracting key features to predict the weight of pigs without the need for any additional feature engineering or preprocessing steps. Zhang et al. [94] proposed a method for rapidly and automatically estimating the weight and body shape of pigs using a deep learning model. By comparing different convolutional neural network models, it was found that the modified Xception model performed best on the test data and was able to complete the estimation task with high accuracy. He et al. [95] used a dual-stream cross-attention visual Transformer to predict the weight of pigs based on RGB and depth images. The combination of the two modalities provides a complementary representation of spatial body information for pigs, resulting in a mean absolute error of 3.237 kg. Chen et al. [96] proposed a method for live pig weight using a multi-layer radial basis function (RBF) network. The network autonomously learns and predicts the weight of live pigs by taking into account body parameters such as body length, height, and width. The results show that this network structure has high accuracy and stability in predicting live pig weight. This method reduces reliance on two-dimensional area parameters of live pigs, minimizes the impact of environmental conditions, lighting, and changes in pig posture on the prediction system’s stability, and offers a practical technological approach for smart agriculture and pig health management. 

Liu et al. [97] proposed a method for estimating pig weight based on 3D hybrid filtering and convolutional neural networks. This approach combines statistical filtering and DBSCAN clustering techniques to accurately segment pigback images, while using voxel subsampling to improve real-time efficiency. By utilizing parameters from pigbacks and convolutional neural networks, the method achieves precise weight estimation, with mean absolute error (*MAE*), mean absolute percentage error (*MAPE*), and root mean squared error (*RMSE*) of 12.45 kg, 5.36%, and 12.91 kg, respectively. In comparison to existing 2D and 3D weight estimation methods, this approach simplifies equipment configuration, reduces data processing complexity, and maintains estimation accuracy—providing an effective monitoring solution for precision pig farming management. 

Tan et al. [98] proposed a method for estimating the weight of pigs based on a dual-stream fusion network and ConvNeXtV2, utilizing RGB-D data for precise measurements. By integrating RGB and depth images, the algorithm accurately estimated the weight of moving pigs in dynamic farm environments. The method employed advanced feature extraction and fusion techniques, achieving lower Mean Absolute Error and Mean Absolute Percentage Error. Liu et al. [99] introduced an unconstrained deep learning-based model for pig body mass estimation (PMEM-DLWC), utilizing Mask R-CNN for pig instance segmentation, Keypoint R-CNN for keypoint detection, and an enhanced ResNet for estimating pig body mass. This model utilizes image processing and deep learning technologies to accurately and rapidly estimate pig body mass in open environments without restricting the movement of the pigs.

Experimental results showed that the model achieved a root mean squared error (*RMSE*) of 3.52 kg on the test set, demonstrating high estimation accuracy and real-time performance. This suggests significant potential for adjusting breeding plans and enhancing production efficiency. In their study, Liu et al. [100] introduced a non-contact weight and body measurement model based on dorsal point cloud data of pigs. The model captures three-dimensional point clouds with a depth camera and utilizes a convolutional neural network combined with a multi-head attention mechanism to predict pig weight. Additionally, the incorporation of RGB information as supplementary features has been shown to enhance prediction accuracy. Compared to traditional manual measurement methods, this model presents potential advantages in reducing errors, increasing efficiency, and promoting animal welfare. Xie et al. [101] used a new method based on improved Mask R-CNN to predict the weight of live pigs. They used ResNeSt as the backbone network, combined with FPN and an attention mechanism to improve image segmentation accuracy. They collected back-depth images of 132 pigs through a depth camera, extracted five key body size parameters, including back area, length, width, average depth, and eccentricity, and trained a weight regression model with these parameters.

Paudel et al. [102] proposed a method based on 3D convolutional neural networks to predict pig weight using point cloud data. A stereo depth camera, Intel Real Sense D435, was utilized to capture 3D videos of freely walking fattening pigs, and the PointNet framework was employed for training and validating models on 1186 point clouds. The experimental results revealed that the PointNet regression model achieved a coefficient of determination of 0.94 on the test point clouds, with a validation root mean squared error (*RMSE*) of 6.79 kg and a test *RMSE* of 6.88 kg. These findings demonstrate the strong potential of deep learning in accurately predicting pig weight on point sets, even with a limited training dataset. This study validates the feasibility of using deep learning to predict the weight of farm animals based on point sets. At the same time, it also highlights the necessity of having larger datasets to ensure the most accurate predictions. 

Table 8 shows the estimation of pig weight using deep learning methods, summarizing parameters such as image acquisition devices, weight estimation methods, and the number of pigs. With the progress of technology, the use of deep learning methods for estimating pig weight is increasing, and compared with Table 7, the number of pigs participating in the experiment is also increasing.

When utilizing machine learning and deep learning algorithms to predict pig weight, it has been observed that deep learning algorithms outperform other methods in terms of prediction accuracy. This is evidenced by the higher correlation coefficients and lower root mean squared errors exhibited by the deep learning algorithms. These findings suggest that, with adequate data support, deep learning algorithms may offer superior performance for pig weight prediction tasks and demonstrate strong generalization capabilities, rendering them suitable for implementation across various pig farms. However, it should be noted that employing deep learning methods for weight estimation necessitates a substantial amount of labeled data for training. This often entails significant human and material resources in the data collection and labeling process.

## 6. Discussion

### 6.1. Current Main Challenges

In recent years, domestic and foreign researchers have made numerous improvements and innovations in the field of automatic livestock body size measurement and weight estimation, achieving fruitful results. However, this technology still requires enhancement in aspects such as real-time performance, accuracy, universality, and automation level [99,100]. When researching livestock body size measurement and weight estimation technology, the following key issues need attention:

Regarding data acquisition: When collecting video data of pigs on a farm, complex background environment changes like lighting and shadows, as well as obstructions from railings and buildings, can affect data quality and impede the accurate detection of key body parts of pigs [103]. Moreover, using multiple sensors for data collection increases costs and restricts the commercial promotion and application of automatic livestock weight estimation technology [45].

In terms of three-dimensional point cloud data: The measurement of pig body size and weight estimation are mostly based on three-dimensional point clouds. Although they can offer more position and detail information with high precision, the large volume of data often leads to feature redundancy, consumes a significant amount of calculation time, demands high computing power, and thus affects the efficiency of information transmission and data processing. Additionally, it cannot ensure the real-time acquisition of livestock three-dimensional point cloud information [104].

Concerning point cloud registration: The coincidence rate of multi-view pig point cloud data is low, and existing point cloud registration algorithms tend to lead the registration into a locally optimal solution. Since livestock are non-rigid objects, their body movements can cause asynchrony in multi-viewpoint cloud data, further increasing the difficulty of point cloud registration.

In the aspect of pig weight estimation: Many studies on pig weight estimation lack robustness and universality, with algorithms relying on data from specific scenarios.

Regarding data acquisition efficiency: When collecting large-scale livestock data, although the data acquisition effect is good when livestock are stationary, controlling livestock behavior can easily trigger stress reactions, and the acquisition efficiency is low, which is not conducive to the automation and large-scale application of body size measurement technology.

In terms of model generalization ability: In actual application scenarios, although the model shows good generalization in an experimental environment, its generalization ability needs to be further verified when dealing with pigs of different breeds and growth dates [53].

### 6.2. Future Development Trends

With the development of three-dimensional image reconstruction technology, the existing problems in livestock body size measurement and weight estimation are expected to be effectively solved. The future development directions in this field are as follows:

In order to solve the problems of illumination, shadow, and occlusion that farms encounter when collecting video data of pigs, multi-sensor fusion technology can be employed. This technology can be combined with image enhancement and illumination normalization algorithms to enhance data quality. Specifically, multiple cameras can be arranged to capture pig videos from diverse angles. Meanwhile, high dynamic range imaging (HDR) technology can be utilized to mitigate the impact of illumination variations. Additionally, deep learning models like convolutional neural networks (CNNs) can be applied to perform illumination normalization and shadow suppression on images, thereby improving the detection accuracy of the key body parts of pigs.

With regard to the issue of sensor cost, it is possible to reduce the cost by optimizing algorithms and choosing sensors with high cost-performance ratios. Meanwhile, an automated data analysis platform can be developed to lessen the dependence on manual operations. In this way, on the premise of not sacrificing data quality, a balance can be achieved between cost-effectiveness and technology promotion. As the three-dimensional reconstruction technology advances, the single-image three-dimensional reconstruction technology can be employed for three-dimensional reconstruction. The collection of a single image is more convenient as only a camera is required to gather data. Its cost is lower than that of collecting three-dimensional point clouds using multiple-depth cameras, and the image reconstruction method is simpler and faster, which reduces the preprocessing steps and calculation errors of three-dimensional point clouds.

In response to the issues of low coincidence rate of multi-view pig point cloud data, proneness to falling into local optimal solutions, and data asynchrony resulting from the movement of livestock as non-rigid objects, the following solutions can be implemented. Firstly, scans are gradually aligned to the standard coordinate system through an incremental process. Inspired by image-based three-dimensional reconstruction, a sparse scan graph is established. Secondly, a second-order spatial compatibility metric is introduced, and the model generation and selection in the classic RANSAC method are re-explored to achieve rapid and robust point cloud registration. Additionally, for the registration challenges of non-rigid objects, deep learning technology can be utilized to extract point cloud features and combined with a consistency decision algorithm to obtain initial correspondences, thereby enhancing the accuracy and robustness of registration. Finally, through global optimization strategies such as simulated annealing or genetic algorithms, the situation where the algorithm stops when iterating to a local optimal solution is avoided, enabling it to jump out of the local optimal solution and find the global optimal solution. By integrating these methods, the registration quality of multi-view pig point cloud data can be effectively enhanced, the likelihood of falling into local optimal solutions can be reduced, and the problem of data asynchrony caused by livestock movement can be resolved.

To enhance the robustness and universality of pig weight estimation, multi-source data fusion technology can be employed. By integrating computer vision and deep learning models, multiple body size parameters, including body length and chest circumference, can be comprehensively utilized for predicting weight. At the same time, through data augmentation, transfer learning, and cross-scene verification, the generalization ability of the model can be improved. Moreover, with the support of a real-time monitoring and management system, data-driven accurate weight estimation can be realized, reducing the dependence on data in specific scenarios and ensuring accurate weight estimation results in diverse breeding environments.

In the aspect of optimizing the collection environment and process, the layout design of the farm can be remodeled to construct a naturally guided collection environment. Sound and light can be utilized to guide livestock to be distributed within the collection area. Meanwhile, an intelligent collection process can be devised, involving batch collection and the implementation of a dynamic collection strategy. Regarding technological innovation and application, non-contact collection technologies such as laser scanning and three-dimensional modeling, machine vision and deep learning, as well as sensor fusion and intelligent analysis, can be adopted. Multiple types of sensors can be integrated, and big data analysis and artificial intelligence technologies can be employed to handle data. In terms of personnel training and management, collectors can receive professional training to enhance their skills. Team collaboration and communication can be reinforced. Additionally, awareness of animal welfare can be fostered. Publicity and educational activities can be launched, and an incentive mechanism can be established. Through these measures, the efficiency of large-scale livestock data collection can be enhanced, and the automated and large-scale development of body size measurement technology can be propelled.

To address the issue that the generalization ability of the model in practical application scenarios requires verification when it comes to pigs of different breeds and growth dates, measures can be taken from three aspects: data augmentation and diverse collection, model optimization, and improvement, as well as real-time monitoring and feedback adjustment. In the area of data augmentation and diverse collection, it is essential to expand the dataset by gathering data on pigs belonging to different breeds and covering the entire growth cycle. Additionally, techniques such as image augmentation and data synthesis should be applied to enhance data diversity. For model optimization and improvement, a multi-model fusion strategy like ensemble learning can be employed. Moreover, pre-trained models can be utilized for transfer learning and fine-tuned according to specific breeds and growth stages. In terms of real-time monitoring and feedback adjustment, an online learning mechanism can be established to monitor the model’s prediction results in real-time and collect feedback. Simultaneously, a variety of evaluation metrics can be used, and the model can be validated in actual scenarios. Through these steps, the generalization ability of the model for pigs of different breeds and growth dates can be effectively enhanced.

### 6.3. Feasibility Analysis of Single-Image 3D Reconstruction

Current research predominantly uses point cloud generation methods from depth cameras to estimate the body dimensions and weight of livestock. However, this approach introduces several issues, including the relatively high cost of depth cameras, significant influence from the data collection environment, and limited shooting angles. With the emergence of technologies such as neural radiance fields and 3D Gaussian splatting, 3D reconstruction methods based on multi-view or single-view images are becoming increasingly sophisticated. 

Hong et al. [105] proposed the first Large Reconstruction Model (LRM), capable of generating a 3D model of an object from a single input image in just 5 s. This model performs 3D reconstruction of an object based on a single image input, utilizing a high-capacity model and extensive training data to achieve versatility and produce high-quality 3D reconstructions from various test inputs, including real-world field captures and images created by generative models.

Since LRM’s approach does not incorporate the geometric priors of the three-planar components into its architecture, and due to the limited size of 3D data and slow training, it often results in poor training quality. Therefore, Wang et al. [106] introduced the convolutional reconstruction model (CRM), a high-fidelity feed-forward single-image to 3D generative model. Recognizing the constraints imposed by sparse 3D data, it is essential to integrate geometric priors into network design. CRM is based on the key observation of the spatial correspondence of six orthogonal images displayed in tri-planar visualization. Firstly, it generates six orthogonal view images from a single input image. Then, these images are fed into a convolutional U-Net, which leverages its powerful pixel-level alignment capabilities and ample bandwidth to create high-resolution tri-planes. Additionally, CRM utilizes flexicubes as a geometric representation, facilitating direct end-to-end optimization of textured meshes.

With the advancement of technology, some scholars have applied single-view or multi-view reconstruction to agriculture. Hu et al. [107] utilized neural radiance fields, focusing on two fundamental tasks in plant phenotypic analysis: the synthesis of new viewpoint images from two-dimensional images and 3D reconstruction of plant models. They introduced a novel plant phenotype dataset containing real plant images from production environments aimed at comprehensively exploring the advantages and limitations of NeRF in the agricultural sector.

The experimental results have demonstrated the strong performance of NeRF in synthesizing new viewpoint images. Moreover, Zhu et al. [108] extended this research by measuring reconstructed plant phenotypic parameters and proposing a system for three-dimensional modeling and phenotypic parameter acquisition of seedling crops based on neural radiance fields. This demonstrates the potential of NeRF in enhancing the capabilities of three-dimensional modeling and phenotypic parameter acquisition in agricultural applications.

Using a smartphone, they captured RGB images from different perspectives, and the NeRF algorithm was employed to construct 3D models. Based on this, algorithms like circle fitting and triangulation were used to accurately measure plant phenotypic parameters such as plant height, stem thickness, and leaf area. The method extracted stem height and stem thickness of pepper seedlings with determination coefficients (R^2^) of 0.971 and 0.907, respectively, and root mean square errors (*RMSE*) of 0.86 cm and 0.017 cm, respectively. For leaf area extraction in different seedling stages, the $R^{2}$ ranged from 0.909 to 0.935, and *RMSE* ranged from 0.75 to 3.22 cm^2^, indicating high measurement accuracy.

The previously mentioned methods illustrate the progress and viability of 3D reconstruction techniques in plant reconstruction and phenotypic estimation. Consequently, it is viable to pursue three-dimensional reconstruction of livestock using neural radiance fields and Gaussian splatting techniques, achieved by capturing multi-view or single-view images of the animals. This approach can then be utilized to estimate the body dimensions and weight of the livestock. Compared with pigs, the epidemic prevention levels of beef cattle and sheep are relatively low. Moreover, pigs cannot obtain the parameters for estimating body weight through a fixed collection channel like cattle or sheep, and the difficulty of image collection is relatively high. Therefore, the method of developing single-image 3D reconstruction of pigs is applicable to almost all livestock.

## 7. Conclusions

The estimation of pig weight based on computer vision collects pig data in a non-contact and non-stress manner and utilizes image processing and three-dimensional point cloud technology to estimate the weight of live pigs, achieving remarkable research progress. This research reviews the weight estimation methods based on images and three-dimensional point clouds, analyzes the advantages and disadvantages of various research methods, and explores the key aspects and development directions of future research. 

In terms of technical means and progress, it evolved from using visible light cameras in the early stage to employing binocular cameras later, combining algorithms to detect body size parameters for weight estimation. Subsequently, with the aid of depth cameras or three-dimensional scanners to construct three-dimensional models, after preprocessing, body size parameters are calculated, and a weight prediction model is established.

Regarding the effect of technical improvement, compared with traditional machine learning algorithms, deep learning algorithms exhibit better performance in prediction accuracy, having a higher correlation coefficient and a lower root mean square error. They can extract key information from high-dimensional complex features, thus obtaining more accurate and reliable results.

Although some progress has been made, numerous challenges still exist. The research results of livestock body size detection and weight estimation are compared and analyzed, and the challenges such as high cost, low automation level, and poor universality faced by current livestock body size measurement research, as well as the development trends and feasibilities in this field in the future, are presented.

In conclusion, the estimation of pig weight based on machine vision is still in the research phase, and there are many unresolved problems, including differences in the body shape, breed of pigs, and breeding methods among different farms. Conducting a series of in-depth research on these technical problems, further improving and optimizing point cloud registration algorithms, data acquisition methods, point cloud registration algorithms, and single-image three-dimensional reconstruction technology, and accelerating the informatization process of the livestock industry are the future research directions.

## Figures and Tables

**Table 1:** Estimation of body weight based on body size parameters.

Number of Pigs	Days	Weight Estimation Methods	Weight Estimation Parameters	*R* ^2^	References	Year
279	42–48	Multiple linear regression	Body length, chest girth, etc.	0.93–0.96	Panda [20]	2021
56	—	Multiple linear regression	Chest girth, body length, etc.	0.955	Machebe and Ezekwe [21]	2010
264	15–56	Path analysis	Front leg height, body length, etc.	0.7359	Banik [22]	2012
47	1–49	Multiple linear regression	Body height, heart girth, etc.	0.86	Oluwole [23]	2014
193	110–230	Multiple linear regression	Body length, heart girth, etc.	0.903	Al Ard Khanji [24]	2018

**Table 2:** Comparison of the content of related studies.

Research	Progress	Technical Approach	Challenge	References (Year)
Pig welfare in precision animal husbandry	Precision animal husbandry techniques in pig welfare monitoring and enhancement	Machine learning, sensors	Cost of technology, data privacy, accuracy of remote monitoring, etc.	Benjamin [4] (2019)
Deep learning applications for livestock behavior recognition	Deep learning in recognizing livestock behavior	Deep learning, behavioral recognition	Data imbalance, complex farming environments, etc.	Rohan [14] (2024)
Precision management of livestock	Techniques for non-contact acquisition of livestock phenotypic data	3D reconstruction techniques, body size acquisition techniques	Lack of accurate 3D reconstruction models, inefficient point cloud acquisition methods, etc.	Ma [18] (2024)
Livestock body measurements	Advances in body measurements of domestic animals	Depth cameras, 2D cameras, deep learning	High cost of equipment, large volume of data, etc.	Ma [19] (2024)
Pig weight measurement	Developments and challenges in non-contact pig weight estimation techniques	Image processing, machine learning	Pig movement, ceiling height, low lighting intensity, etc.	Bhoj [25] (2022)
Animal weight measurement	Comparing the development of different animal weighing techniques	Traditional image weighing, deep learning weighing, feature parameter extraction methods	Data quality, model generalization capabilities, real-time performance, etc.	Zhao [26] (2023)

**Table 3:** Statistics of Pearson’s correlation coefficient of weight estimation parameters.

	Weight	Body Length	Body Width	Body Height	Chest Girth	Chest Depth	Abdominal Length
Weight	1	0.750	0.683	0.788	0.926	0.728	0.328
Body length	—	1	0.398	0.632	0.715	0.653	0.442
Body width	—	—	1	0.486	0.683	0.423	0.131
Body height	—	—	—	1	0.773	0.733	0.176
Chest girth	—	—	—	—	1	0.699	0.225
Chest depth	—	—	—	—	—	1	0.222
Abdominal length	—	—	—	—	—	—	1

**Table 4:** Estimation of body weight based on image parameters.

Number of Pigs	Number of Cameras	Camera Angle	Effect of Light	Weight Estimation Parameters	Weight Estimation Methods	Error	References (Year)
15	1	Aerial view	Manual screening	Projection area	Linear regression	5%	Schofield (1999) [36]
12	1	Aerial view	Adding an external light source	Projection area, height	Multiple linear regression	0.8%	Minagawa (2001) [37]
25	1	Aerial view	—	Body length, body width, body area	Multiple linear regression	1.34 kg	Doeschl (2004) [38]
24	1	Aerial view	—	Projection area, body length	Multiple linear regression	4.1%	Wang (2006) [39]
50	2	Side view and aerial view	Histogram equilibrium	Projection area	Linear regression	2.8%	Yang (2005) [40]
24	1	Aerial view	Edge detection	Projection area	Nonlinear regression	4.1%	Wang (2008) [41]
88	1	Aerial view	—	Chest girth, body length	ANN	6.243%	Kaewtapee (2019) [42]
150	2	Side view and aerial view	—	Body length, height, etc.	Multiple linear regression	3.4%	Wu (2020) [43]
35	1	Aerial view	Image enhancement	Body length, body width, etc.	Multiple linear regression	1.18 kg	Banhazi (2011) [44]
265	3	Aerial view and side view	—	Body length, projection area, etc.	Nonlinear regression	—	Gaganpreet (2023) [45]
52	1	Aerial view	—	Body length, chest girth, back area	Multiple linear regression	2.39 kg	Cunha (2024) [46]
800	1	Aerial view	—	Back area	Deep learning	3.11 kg	Wan (2024) [47]

**Table 5:** Measurement of pig body size parameters based on 3D point cloud.

Number of Pigs	Days	Number of Cameras	Camera Angle	Parameters	Error	References (Year)
25	—	3	Side view and aerial view	Body length, body width, etc.	<4%	Yin (2022) [48]
4	147–154	1	—	Body length, body width, hip width, etc.	<16 mm	Wang (2017) [57]
10	141–149	1	Askew view	Height, chest girth, abdominal circumference, etc.	<8%	Wang (2018) [58]
25	—	3	Side view and aerial view	Body length, chest width, etc.	<5%	Yin (2019) [59]
—	—	2	Side view	Body length, shoulder width, hip width, etc.	<4%	Guo (2014) [60]
—	—	2	Side view	Body length, body width, hip height, etc.	<4%	Qin (2020) [61]
10	130–220	—	—	Body length, chest girth, shoulder height, etc.	<8%	Guo (2017) [62]
25	—	3	Side view and aerial view	Body length, body width, etc.	<6%	Hu (2023) [63]
20	175–224	2	Adjustable	Body width, hip width height, etc.	<11%	Wang (2018) [64]
13	—	3	Side view and aerial view	Chest girth, body length, etc.	<21 cm	Du (2022) [68]
13	—	3	Side view and aerial view	Body length, chest girth, chest depth, etc.	<11 cm	Luo (2023) [69]
10	—	5	Side view and aerial view	Body length, chest girth, etc.	<5%	Lei (2024) [70]

**Table 6:** Estimating live pig weight based on point clouds.

Number of Pigs	Number of Cameras	Camera Angle	Weight Estimation Parameters	Weight Estimation Methods	*MAE*/kg	*RMSE*/kg	*R* ^2^	References (Year)
251	1	Aerial view	Body length, rib width, etc.	Nonlinear regression	—	1.8	0.98	Franchi (2023) [8]
234	1	Aerial view	Volume projection	Linear regression	—	—	0.9907	Condotta (2018) [71]
20	1	Aerial view	—	Deep learning	0.644	—	—	Cang (2019) [72]
29	1	Aerial view	—	Deep learning	6.366	—	—	He (2021) [73]
655	1	Aerial view	Projection area, volume projection, etc.	Multiple linear regression	—	—	0.86	Arthur (2019) [74]
50	1	Aerial view	Body length, height, shoulder width, etc.	Multiple linear regression	2.9617	2.616	0.958	Li (2022) [75]
15	1	—	Body length, chest girth, HOG feature	Multiple linear regression	—	10.702	—	Na (2023) [76]
733	1	Handhold	Body length, body width, chest girth	Nonlinear regression	9.25	12.3	—	Nguyen (2023) [77]
70	4	Side view and aerial view	Body width, height, round, etc.	Deep learning	4.89	8.6899	0.9532	Kwon (2023) [78]
582	1	Aerial view	Volume projection	Linear regression	2.84	—	—	Selle (2024) [79]

**Table 7:** Weight estimation based on traditional methods.

Number of Pigs	Image Acquisition Equipment	Camera Angle	Weight Estimation Parameters	Weight Estimation Methods	*R* ^2^	References (Year)
40	Visible light camera	Aerial view	Projection area	Linear regression	0.9663	Kashiha (2014) [80]
10	Binocular camera	Aerial view	Body length, shoulder height, etc.	Linear regression	0.99	Shi (2016) [81]
358	—	—	Body length, chest girth etc.	Linear regression	0.89	Sungirai (2014) [82]
—	—	—	Body length, chest girth	Linear regression	0.93	Alenyoregue (2013) [83]
183	—	—	Body length, chest girth, etc.	Linear regression	0.90	Al Ard Khanji (2016) [24]
61	Visible light camera	Aerial view	Projection area, round, etc.	Linear regression	0.9925	Wang (2008) [12]
73	Visible light camera	Aerial view	Mean distance between centers of mass, round, etc.	Nonlinear regression	—	Wongsriworaphon (2015) [85]
513	Depth camera	Aerial view	Curvature, misalignment, etc.	Nonlinear regression	0.790	Jun (2018) [86]
78	Depth camera	Side view and aerial view	Body length, chest girth, etc.	Multiple linear regression	0.9942	Pezzuolo (2018) [87]
23	Depth camera	Side view and aerial view	Body length, body width, etc.	Multiple linear regression	—	Kwon (2022) [9]
340	—	—	Body length, chest girth, shoulder height, etc.	Multiple linear regression	—	Ruchay (2022) [88]
279	—	—	Body length, chest girth, etc.	Multiple linear regression	0.9131	Preethi (2023) [89]
18	Visible light camera	Aerial view	Back area	Linear regression	0.99	Tu (2023) [90]
39	Depth camera	Aerial view	Back area, body length, etc.	Multiple linear regression	0.995	Jiang (2024) [91]
479	Depth camera	Side view and aerial view	Point cloud volume	Linear regression	0.921	Lin (2024) [92]

**Table 8:** Weight estimation based on deep learning.

Image Acquisition Equipment	Weight Estimation Methods	Number of Pigs	*MAE*/kg	*RMSE*/kg	*MAPE*/%	*R* ^2^	References (Year)
Depth camera	Deep learning	400	—	3.8	3.9	0.397	Meckbach (2021) [93]
Depth camera	Deep learning	239	1.16	1.53	1.99	0.9973	Zhang (2021) [94]
Depth camera	Deep learning	—	3.237	5.993	4.082	0.742	He (2023) [95]
Depth camera	Deep learning	4721	1.85	5.74	1.68	0.63	Chen (2023) [96]
Depth camera	Deep learning	—	12.45	12.91	5.36	—	Liu (2023) [97]
Depth camera	Deep learning	198	2.856	4.082	2.383	0.901	Tan (2023) [98]
Binocular camera	Deep learning	117	—	3.52	2.82	—	Liu (2023) [99]
Depth camera	Deep learning	258	11.81	11.552	4.81	—	Liu (2024) [100]
Depth camera	Deep learning	132	2.96	3.95	8.45	0.987	Xie (2024) [101]
Depth camera	Deep learning	249	—	6.88	—	0.94	Paudel (2024) [102]

## Data Availability

The original contributions presented in the study are included in the article. Further inquiries can be directed to the corresponding authors.
